# Characterization of acetovanillone degradation in wild-type and engineered *Rhodococcus aromaticivorans* RHA1

**DOI:** 10.1128/aem.02522-25

**Published:** 2026-03-20

**Authors:** Anne T. Lalande, Megan E. Wolf, Logan D. Robeck, Lindsay D. Eltis

**Affiliations:** 1 Department of Microbiology and Immunology, Life Sciences Institute, The University of British Columbia 468813, Vancouver, BC, Canada; Danmarks Tekniske Universitet, Kgs. Lyngby, Denmark

**Keywords:** lignin, acetovanillone, co-metabolism, biocatalysis

## Abstract

There is considerable interest in harnessing bacterial catabolic activities to valorize lignin-derived compounds. One such class of compounds, hydroxyphenylethanones (HPEs), includes acetovanillone (AV), 4-hydroxyacetophenone (HAP), and acetosyringone (AS). Despite occurring in various industrial streams of lignin, there are few reports on their microbiological degradation. Here, we report that *Rhodococcus aromaticirovans* RHA1 cometabolizes, but does not grow on, AV and HAP, converting them to 3,4-dihydroxyacetophenone (3,4-DHAP), a catechol with pharmacological properties. Molecular genetics studies revealed that AgcAB, a cytochrome P450 alkylguaiacol *O*-demethylase, catalyzes AV *O*-demethylation and AphAB, an alkylphenol hydroxylase, catalyzes HAP hydroxylation. Additionally, AV induced the expression of AgcA and the alkylcatechol extradiol dioxygenase, AphC. The specificity (*k*_cat_/*K*_M_) of AgcAB for AV was ~7% that of 4-propylguaiacol, while AphC cleaved 3,4-DHAP at ~8% the rate of 4-methylcatechol. We also compared the activity of HPE catabolic pathways from three strains by integrating their genes into RHA1. In resting cell assays, a strain expressing the *hpe* genes of *Rhodococcus rhodochrous* GD02 converted AV 43- and 97-fold faster than strains expressing pathways from *Actinomadura macra* sp. NBRC-14102 and *Sphingobium lignivorans* SYK-6, respectively. All three pathways preferentially converted AV and HAP over AS. However, the strain expressing the SYK-6 pathway had the highest relative activity for AS, 18% its activity on AV. The GD02 *hpe* genes enabled the growth of RHA1 on HPEs, and it grew faster on AV than on GD02. Overall, this work provides insight into the substrate preference of aromatic catabolic enzymes, highlights the value of cometabolism for biocatalysis, and facilitates the engineering of microbial cell factories to valorize HPEs.

**IMPORTANCE:**

As an abundant polymer in plants, lignin represents a renewable alternative to petroleum as a feedstock in the manufacture of chemicals. Strategies to convert lignin into higher-value compounds couple chemical and biological catalysis, where engineered microorganisms transform chemocatalytically generated mixtures of lignin-derived aromatic compounds, or LDACs. The development of microbial biocatalysts depends on the characterization and engineering of pathways that degrade LDACs. In this study, we identified a pathway that partially degrades an important class of LDACs in *Rhodococcus aromaticivorans* RHA1, an organism with considerable biocatalytic potential. The study includes expanding the known substrate range of previously characterized pathway enzymes. We also compare three homologous catabolic pathways, contrasting their efficiency and potential in biocatalysis. These findings advance our understanding of the catabolism of aromatic compounds and facilitate the engineering of microbial cell factories to sustainably transform lignin into platform chemicals.

## INTRODUCTION

Lignin is a complex biopolymer that primarily confers structural integrity to terrestrial plants (1). As the most abundant reservoir of aromatic compounds on Earth, it is an important source of carbon and energy for environmental microorganisms. In Nature, lignin degradation by biological or geochemical processes releases aromatic monomers, which serve as growth substrates for a wide variety of bacteria (2–4). The catabolic processes and the enzymes that enable this growth are critical in the global carbon cycle and have broad-ranging biotechnological applications, including lignin valorization (5). Indeed, the biocatalytic conversion of lignin streams into higher-value chemicals has been touted as a sustainable alternative to petroleum-based chemical production (5–7). Emergent valorization strategies involve tandem chemo-biological approaches consisting of an initial chemical fractionation step to isolate and depolymerize lignin into aromatic monomers, followed by transformation of monomer mixtures into target products by engineered microbial cell factories (7). Because the composition of the monomer mixture varies greatly with both the biomass source and the fractionation method, microbial biocatalysts must be modular and customizable.

**Fig 1:**
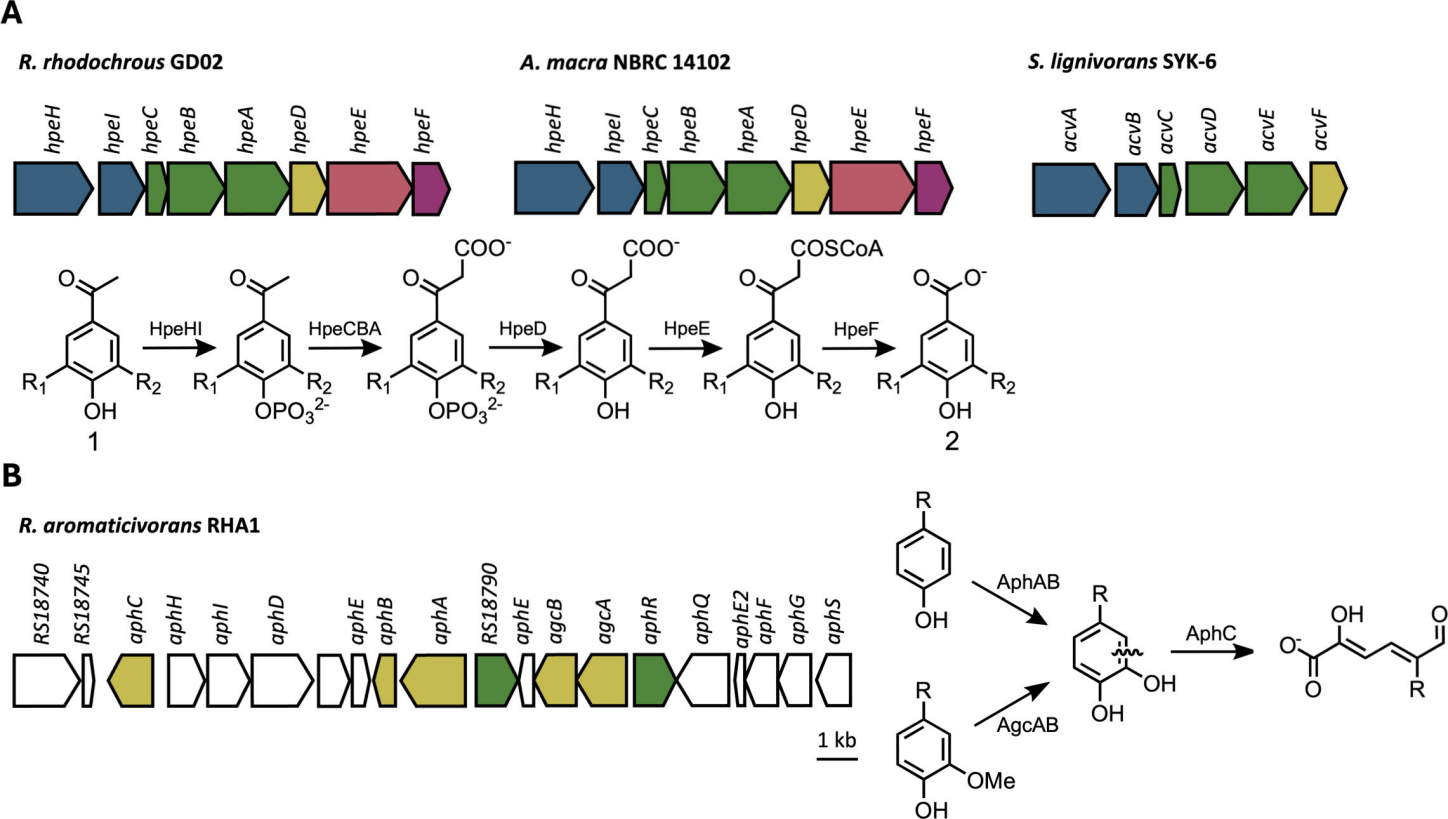
Bacterial degradation of HPEs and alkylated phenols and guaiacols. (**A**) *R. rhodochrous* GD02 (16), *A. macra* NBRC 14102, and *S. lignovorans* SYK-6 (15) encode genes for the catabolism of HPEs. The pathway is shown as proposed in reference 16, although the proposed reaction order is different in reference 15. Homology (>40% amino acid sequence identity) is indicated by color. As shown, the pathway converts AV (R_1_ = H, R_2_= OMe in Compound 1), HAP (R_1_, R_2_ = H in Compound 1), and AS (R_1_, R_2_ = OMe in Compound 1) to vanillate, 4-hydroxybenzoate, and syringate, respectively (Compound 2). (**B**) The gene cluster encoding a degradation pathway for alkylated phenols and guaiacols in RHA1. In this pathway, alkylated phenols and guaiacols are hydroxylated and demethylated, respectively, to the alkylcatechol, which then undergoes *meta*-cleavage (R = alkyl group). Genes discussed in this work are highlighted in yellow, and putative regulators are highlighted in green.

Hydroxyphenylethanones (HPEs) are a class of compounds that frequently occur in monomer streams generated by oxidative catalytic fractionation of lignin, as well as in black liquor generated by Kraft pulping (8–13). Accordingly, there is considerable interest in understanding the bacterial degradation of HPEs, which include acetovanillone (AV), 4-hydroxyacetophenone (HAP), and acetosyringone (AS). A pathway for the catabolism of HPEs was recently elucidated in *Rhodococcus rhodochrous* GD02 (GD02) and *Sphingobium lignivorans* SYK-6 (SYK-6, formerly *Sphingobium sp*. SYK-6 (14–16). Termed the Hpe and Acv pathway in GD02 and SYK-6, respectively, it comprises a two-component kinase, a three-component carboxylase, and a phosphatase (Fig. 1A). In GD02, the *hpe* operon additionally encodes an acyl-CoA ligase and β-keto hydrolase, which catalyze β-elimination such that AV and HAP yield vanillate and 4-hydroxybenzoate, respectively. In SYK-6, enzymes catalyzing the last two steps are encoded elsewhere in the genome (15). The Hpe pathway enables the growth of GD02 on AV and HAP but not AS, while SYK-6 can also grow on AS. A very similar pathway has also been described in *Novosphingobium aromaticivorans* DSM12444, except that catabolism is initiated by MarK, a single-component kinase (17). Interestingly, MarK and HpeHI have higher specific activity for AV than AS, while AcvAB exhibits the opposite preference, consistent with the respective growth phenotype of the host organisms (15, 17, 18). Recently, *Pseudomonas rhizophila* AS1 was isolated for its ability to grow on AS, and this catabolism is thought to be initiated by ring-hydroxylation (19). Although reports of growth on HPEs are limited to these four strains or mutants thereof, clusters encoding various combinations of *hpe* gene homologs have been identified in phylogenetically diverse genomes (17). Interestingly, genes encoding the kinase and carboxylase occurred in a cluster in only 52 of 836 cases, suggesting that the operon structure of the *hpe* genes in GD02 is uncommon. Indeed, Dexter et al*.* identified only four genomes with syntenous *hpeHICBADEF* clusters, with *Actinomadura macra* NBRC 140102 being the only non-*Rhodococcus* (16). The relatively poor understanding of HPE catabolism limits efforts to biologically upgrade oxidatively depolymerized lignin streams.

Members of the *Rhodococcus* genus are attractive biocatalysts due to their extensive aromatic catabolic capabilities as well as their ability to tolerate a variety of stresses and industrially relevant conditions (20, 21). For example, a rhodococcal biocatalyst is used to generate thousands of tons of acrylamide annually (22). Rhodococcal genomes are typically large and encode numerous catabolic pathways, including those enabling growth on manifold aromatic compounds (23). Among rhodococci, strain RHA1, *R. aromaticivorans* (formerly, *R. jostii* [24]) has emerged as a promising biocatalytic chassis due to its considerable catabolic repertoire and its genetic tractability, facilitated in part by the development of toolkits such as Serine integrase-Assisted Genome Engineering (SAGE) (25–28). As is typical of aerobic aromatic-degrading bacteria, RHA1’s catabolism has a convergent architecture, whereby diverse oxygenase-rich upper pathways convert a variety of aromatic compounds into a few key intermediates such as protocatechuate and catechol (26, 29, 30). A small number of lower pathways convert these intermediates, initially via ring-cleavage, into central metabolites (29). For example, growth on 4-alkylguaiacol is enabled by a pathway initiated by a cytochrome P450 *O*-demethylase AgcAB. Alkylphenol catabolism is initiated by a two-component flavin-dependent hydroxylase, AphAB (31, 32). The resulting alkylcatechol, a convergent product of both AgcAB and AphAB, undergoes ring-cleavage by an extradiol dioxygenase, AphC, before ultimately yielding pyruvate and an acyl-CoA in a series of reactions catalyzed by the remaining *aph* gene products (30, 31) (Fig. 1B). Likewise, vanillate and 4-hydroxybenzoate, products of the Hpe pathway, are demethylated and hydroxylated, respectively, to yield protocatechuate, which undergoes *ortho*-cleavage and further degradation via the β-ketoadipate pathway (33, 34). Despite its expansive genome, RHA1 does not contain an Hpe pathway and does not grow on AV.

Herein, we investigated the transformation of HPEs by RHA1. Through molecular genetics and biochemical studies, we identified a cometabolic pathway that is induced by and partially degrades AV and HAP. Our studies established that the transformations are catalyzed by the enzymes encoded by the *agc* and *aph* genes, responsible for the catabolism of alkyl-guaiacols and -phenols. We also used RHA1 as a chassis to compare the *hpe* pathways from GD02, SYK-6, and *A. macra*, revealing their substrate preferences and relative efficiency. Additionally, we established that an RHA1 strain overexpressing the *hpe* genes of GD02 grows on AV and HAP, testing two different expression strategies in the process. Our results are discussed with respect to the bacterial catabolism of HPE and the valorization of these compounds.

## RESULTS

### RHA1 transforms AV and HAP

**Fig 2:**
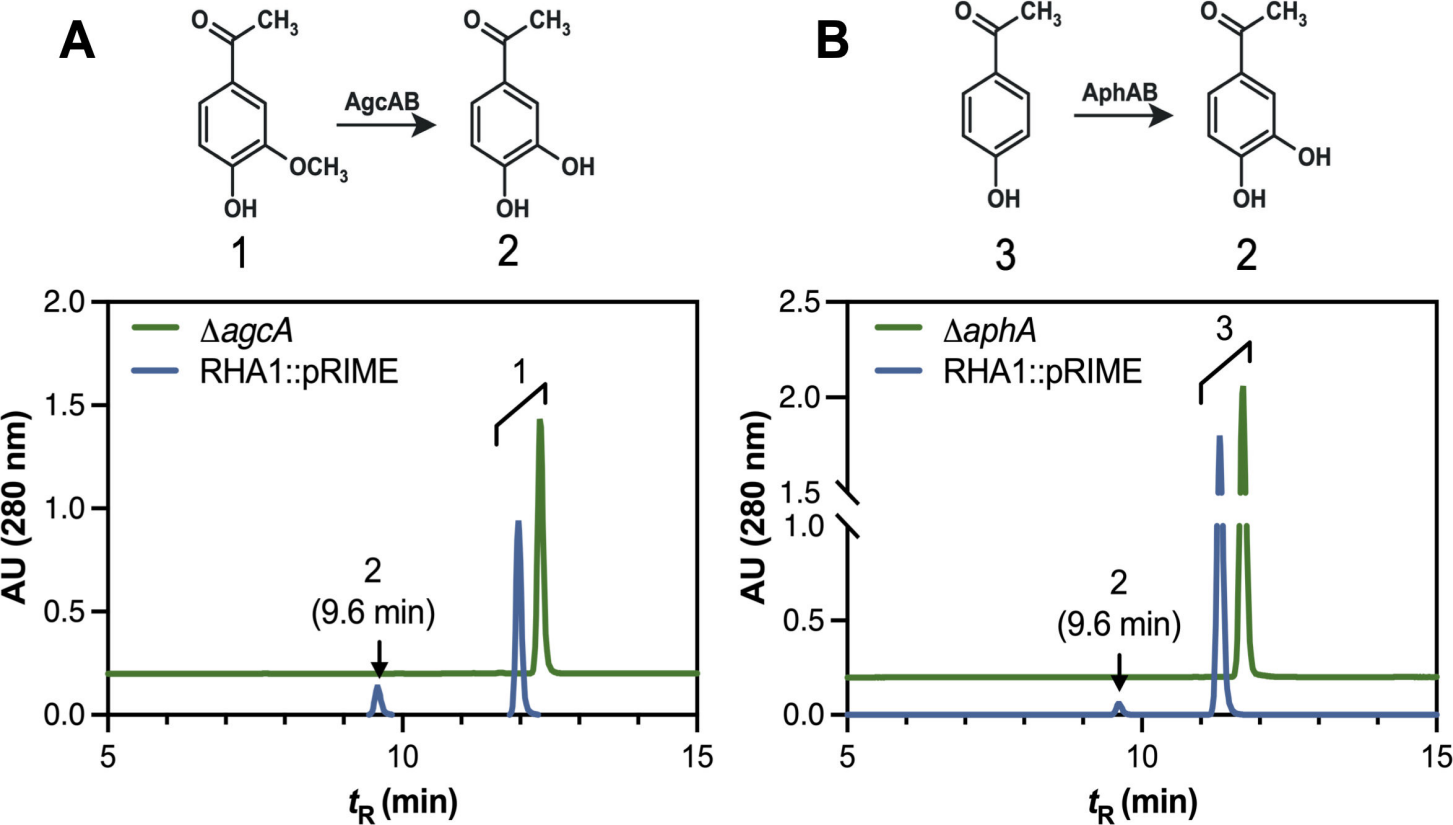
Transformation of AV and HAP to 3,4-dihydroxyacetophenone by resting cells of RHA1. RHA1::pRIME and the indicated mutant strains were incubated in M9 supplemented with 0.1% glucose and 1 mM AV (**A**) or HAP (**B**) in 25 mL flasks at 30°C. Chromatograms are of culture supernatants collected after 3 h for RHA1::pRIME in blue and the Δ*agcA* (**A**) or Δ*aphA* (**B**) mutants in green. The proposed transformations of AV (**1**) and HAP (**3**) to 3,4-DHAP (**2**) are shown. Data are offset for clarity.

Although RHA1 does not grow on HPEs, we sought to determine whether it could transform these substrates. When resting cells of RHA1 harboring an empty integrative vector, pRIME (28), were incubated for 90 min with 1 mM AV or HAP, the substrate was depleted and a new peak appeared at ~9.6 min (Fig. 2). Comparison to an authentic standard identified this peak as 3,4-dihydroxyacetophenone (3,4-DHAP) (Fig. S1). By contrast, resting cells did not detectably transform AS over 3 h (Fig. S2). Due to the structural similarity of AV and HAP to 4-ethylguaiacol (4-EG) and 4-ethylphenol, we hypothesized that the HPEs were transformed by AgcAB and AphAB, respectively. To test this hypothesis, we conducted resting cell assays with the previously generated Δ*agcA* and Δ*aphA* RHA1 mutant strains (31, 35). No 3,4-DHAP was detected when Δ*agcA* was incubated with AV, suggesting that the encoded cytochrome P450 catalyzed the *O*-demethylation of AV (Fig. 2A). Similarly, the Δ*aphA* mutant did not detectably transform HAP to 3,4-DHAP, suggesting that the alkylphenol hydroxylase catalyzed the hydroxylation of HAP (Fig. 2B). By contrast, deletion mutants of a vanillate *O*-demethylase (Δ*vanA* [33]) and a *p*-hydroxybenzoate hydroxylase (Δ*pobA* [34]) both transformed AV and HAP to 3,4-DHAP (Fig. S3).

### AgcAB catalyzes the *O*-demethylation of AV

Having identified *agcA* as likely being required for AV *O*-demethylation in RHA1, we interrogated the ability of the P450 to catalyze this reaction *in vitro* compared to two of its preferred substrates, 4-EG and 4-propylguaiacol (4-PG). The rate of AV depletion by purified AgcAB was approximately nine times lower than that of 4-EG. The coupling efficiency of AV *O*-demethylation to NADH oxidation was 90% ± 10%, compared to 110% ± 7% with 4-EG (Table S3). AgcA’s apparent substrate specificity (*k*_cat_/*K*_M_) for AV was approximately 14 times lower than for 4-PG (Table 1 ; Fig. S4).

**TABLE 1:** Apparent steady-state kinetic parameters of AgcA^*a*^

Substrate	*k*_cat_ (s^−1^)	*K*_M_ (µM)	*k*_cat_/*K*_M_ (mM^−1^s^−1^)	Reference
4-PG	0.146 (0.007)	1.5 (0.3)	97 (12)	(36)
AV	0.143 (0.098)	21 (5)	7 (3)	This study

^*a*^ Apparent steady-state kinetic parameters were measured using 10 mM MOPS pH = 7.2, ionic strength (*I*) = 25 mM at 25°C. The depletion of NADH was measured spectrophotometrically. Error in curve fitting is shown in parentheses.

### Computational modeling of AgcA:AV

To evaluate the ability of the AgcA binding pocket to accommodate an ethanone substituent, we modeled the AgcA_RHA1_:AV complex based on the structure of AgcA_EP4_ from *R. rhodochrous* EP4 in complex with 4-EG (36). When AV was modeled in its thermodynamically favored conformation with the ethanone coplanar to the aromatic ring, the carbonyl clashed sterically with the heme (Fig. S5A; O-C distance of 3.0 Å). Rotating the ethanone substituent around the aryl-acetyl C-C bond to direct the methyl group into the hydrophobic cavity that accommodates 4-EG’s ethyl substituent partially relieved the clash with the heme but positioned the carbonyl oxygen within 2.9 Å of Ile289, which lines the substrate-binding pocket (Fig. S5D). Nevertheless, these steric clashes were not severe enough to preclude AV from binding in a catalytically competent orientation, presumably through small adjustments to residues lining the pocket.

### AphC catalyzes the meta-cleavage of 3,4-DHAP

In RHA1, *agcAB* and *aphAB* occur in a gene cluster encoding a *meta*-cleavage pathway, wherein 4-alkylcatechols are cleaved by AphC (Fig. 1B). Therefore, having established the ability of AgcAB to transform AV, we next investigated the ability of AphC to cleave the transformation product, 3,4-DHAP. RHA1 cultures incubated overnight with AV or HAP developed a bright yellow color, characteristic of the dienolate moiety of *meta*-cleavage products (Fig. S6). Liquid chromatography-mass spectrometry (LC-MS) analysis of the supernatants of these cultures confirmed the presence of a compound with the expected *m/z* value of the 3,4-DHAP *meta*-cleavage product (Fig. S7). No downstream metabolites of the Aph pathway were detected. In an oxygraphy assay, the specific activity of purified AphC (30) for 3,4-DHAP was 30 ± 2 U/mg (air-saturated 20 mM HEPES, pH 8.0, 25°C) as compared to 370 ± 40 U/mg for 4-methylcatechol, a substrate for which the enzyme has a high specific activity (Table 2).

**TABLE 2:** Specific activities of AphC^*a*^

Substrate	Specific activity (U/mg)
4-methylcatechol	370 (40)
3,4-DHAP	30 (2)

^*a*^ Values were measured in air-saturated 20 mM HEPES at 25°C with 1.4 μM (for 3,4-DHAP) or 0.46 μM (for 4-methylcatechol) AphC and 200 μM 4-methylcatechol or 3,4-DHAP.

### AV induces production of catabolic enzymes in RHA1

The *agc* and *aph* genes are upregulated in the presence of alkylguaiacols (31, 35). To determine the extent to which these activities are induced by AV, we first examined the levels of *O*-demethylation activity in cells exposed to AV compared to 4-EG by quantifying the rate of 3,4-DHAP formed from AV. To avoid degradation of the catechol product, these assays were conducted using an ∆*aphC* mutant (31). When cells were exposed to 1 mM AV for 2.5 h, the rate of 3,4-DHAP formation was about three times that of cells exposed to no inducer and approximately 28% that of cells exposed to 1 mM 4-EG (Table 3). In agreement with our previous results, the ∆*agcA* mutant did not produce any 3,4-DHAP regardless of the inducing substrate. To examine the ability of AV to induce AphC activity, we grew RHA1 in the presence of 1 mM AV and measured the ability of cell lysates to cleave 4-ethylcatechol. Extradiol dioxygenase cleavage activity was measured spectrophotometrically. The specific activity of lysates prepared from AV-incubated cells was 230 times that of lysates prepared from succinate-grown cells and 17 times lower than that of lysates of 4-EG-grown cells (Table 2).

**TABLE 3:** Induction of *O*-demethylation and *meta*-cleavage activities in RHA1 following exposure to different aromatic compounds

Inducer^*c*^	*O*-Demethylation (U/g protein)^*a*^	*meta-*Cleavage (U/g protein)^*b*^
4-EG	20.8 (1.3)	38 (4.4)
AV	5.9 (2.8)	2.3 (1.9)
None	2.0 (0.2)	0.01 (0.02)

^*a*^ *O*-Demethylation activity is defined as the rate of 3,4-DHAP production from 1 mM AV by whole cells of Δ*aphC*, where 1 U is defined as 1 μmol 3,4-DHAP produced in 1 min. Cells were grown in LB to mid-log, concentrated in M9, incubated 2.5 h with an inducing substrate, and then 1 h with AV. Rates were measured by HPLC analysis of supernatants at three timepoints. Data were normalized to total cellular protein and represent the average value of biological triplicates with standard deviation.

^*b*^ *meta*-Cleavage activity is defined as the rate 5-ethyl HODA production from 400 μM 4-ethylcatechol by lysates of RHA1, where 1 U is defined as 1 μmol 5-ethyl HODA produced in 1 min. Prior to lysis, cells were grown in 0.1% glucose to mid-log and incubated a further 7 h following the addition of the inducer. Rates were measured spectrophotometrically. Data were normalized to total protein in lysates and represent the average value of triplicates with standard deviation.

^*c*^ In all three conditions, succinate was present as a growth substrate.

### Comparison of three Hpe pathway variants

**Fig 3:**
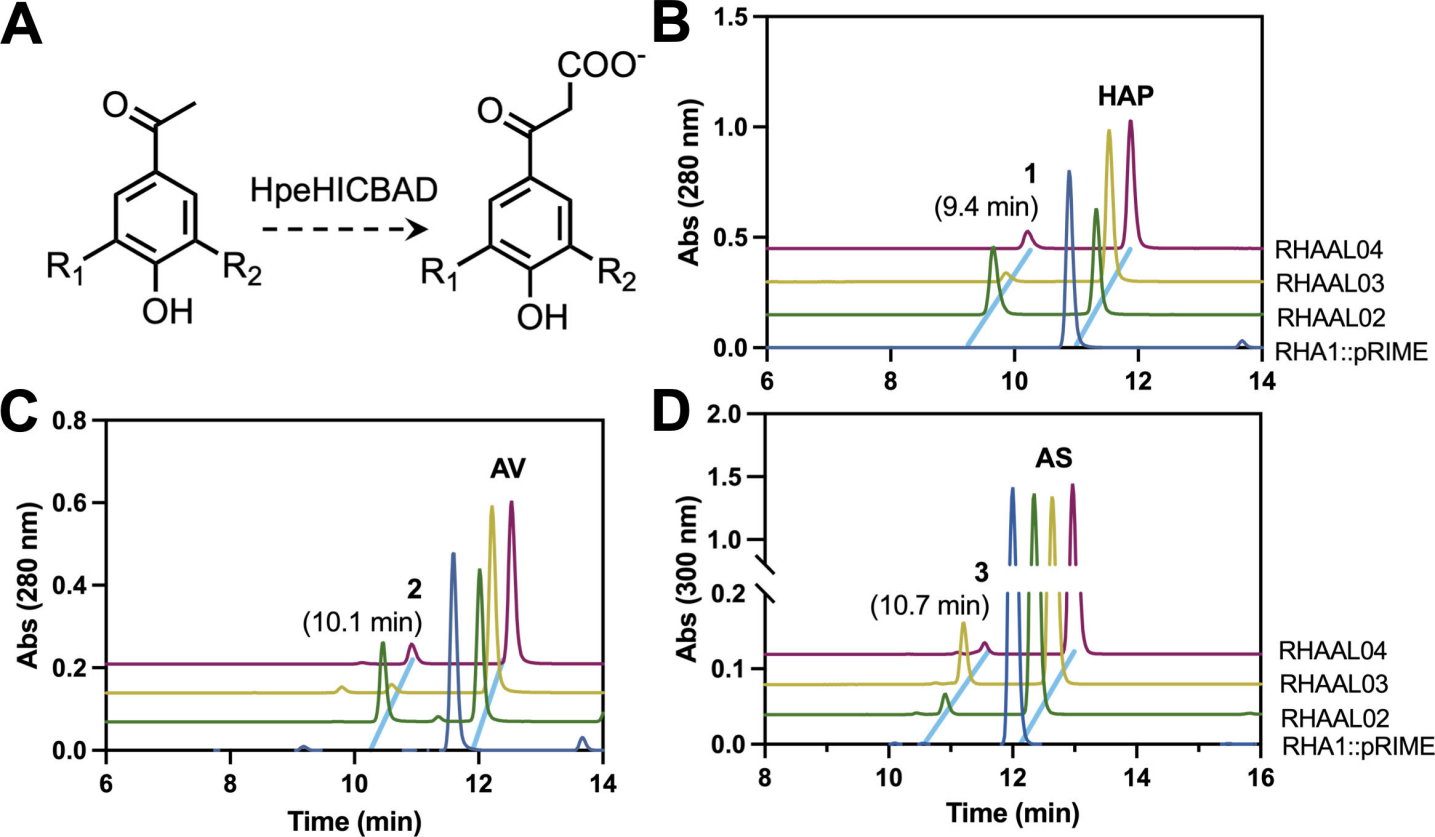
HPE-transforming activity of three pathways expressed in RHA1. The transformation of HPEs to 4-HPβKPs by the successive activities of HpeHI, HpeCBA, and HpeD (**A**). Chromatograms of culture supernatants of the indicated strains incubated with 1 mM HAP (**B**), AV (**C**), or AS (**D**) in M9 supplemented with 0.1% glucose. Strains were incubated at 30°C for 1 h in 24-well plates. For Peak 1, R_1_ and R_2_ = H; for Peak 2, R_1_ = H and R_2_ = OCH_3_; for 3, R_1_ and R_2_ = OCH_3_. The identity of the product peaks was validated using LC-MS (Fig. S9). In panel C, the peaks at 9.6 min are 3,4-DHAP. Strains RHAAL02, RHAAL03, and RHAAL04 contained the first six *hpe*/*acv* genes from GD02, SYK-6, and *A. macra*, respectively. Strain RHA1::pRIME contained the empty pRIME plasmid.

The selection of efficient pathways is critical to biocatalyst design. To date, few Hpe catabolic pathways have been characterized and, for those that have been reported, there has been no direct comparison of their efficacy and substrate preference. To enable such an investigation, we selected pathways from three organisms: GD02, SYK-6, and *A. macra* (8, 15, 37). The three operons are homologous, but that of SYK-6 comprises six genes, while GD02 and *A. macra* comprise eight. As such, we cloned the first six genes of all three operons, encoding the kinase, carboxylase, and phosphatase, into pRIME, an integrative vector, under the control of P_T1_ promoter, a strong constitutive promoter from RHA1 (28). Introduction of the GD02, SYK-6, and *A. macra* genes into RHA1 yielded strains RHAAL02, RHAAL03, and RHAAL04, respectively. In resting cell assays, all three strains transformed HAP, AV, and AS to single major products eluting at 9.4, 10.1, and 10.7 min, respectively (Fig. 3). Small amounts of 3,4-DHAP were also detected in the RHAAL03 and RHAAL04 incubations. LC-MS analysis established that these have the expected *m/z* value of the respective 4-hydroxyphenyl β-ketopropionates (HPβKPs) (Fig. S8).

To compare the activities of the three pathways in RHA1, we measured the rates of formation of the HPβKPs in resting cell assays. To avoid competition by AgcAB and AphAB, cells were incubated for less than 2 h to limit gene induction and enzyme production. Under these conditions, only trace amounts of 3,4-DHAP were detected. The three strains converted AV and HAP to the corresponding HPβKPs at a similar rate for both substrates. The strain containing the GD02 genes had the highest specific activity for AV and HAP, converting AV to 3-methoxy-4-HPβKP at a rate of 107 μmol min^−1^ g^−1^, 97- and 43-fold faster than strains containing the *A. macra* and SYK-6 pathways, respectively. RHAAL03, harboring the SYK-6 pathway, had the highest specific activity for AS, converting it to the 3,5-dimethoxy-4HPβKP at a rate of 0.2 μmol min^−1^ g^−1^, two- and ten-fold faster than RHAAL02 and RHAAL04 (Table 4). LC-MS analysis of the reaction supernatants enabled the detection of other pathway intermediates. Notably, RHAAL03 produced higher relative amounts of phosphorylated HPEs compared to the two other strains (Fig. S9).

**TABLE 4:** Activity of three HPE pathways expressed heterologously in RHA1

Substrate	Pathway activity (U/g protein)^*a*^		
	RHAAL02	RHAAL03	RHAAL04
HAP	97 (7)	1.1 (0.1)	2.3 (0.1)
AV	107 (24)	1.1 (0.5)	2.5 (0.6)
AS	0.09 (0.03)	0.2 (0.03)	0.02 (0.01)

^*a*^ Pathway activity is defined as the rate of (methoxy)−4-HPβKP, formed from 1 mM substrate by whole cells of RHAAL02, RHAAL03, or RHAAL04. Cells were grown in LB to mid-log, concentrated in M9, and incubated with 1 mM substrate. One unit of activity equals 1 μmol HPβKP formed in 1 min, and data are normalized to total cellular protein. Rates were measured by HPLC analysis of supernatants at three or four timepoints. Data represent the average value of triplicates, with standard deviation in parentheses.

### Expression of *hpeHICBADEF* in RHA1 enables growth on AV and HAP

**Fig 4:**
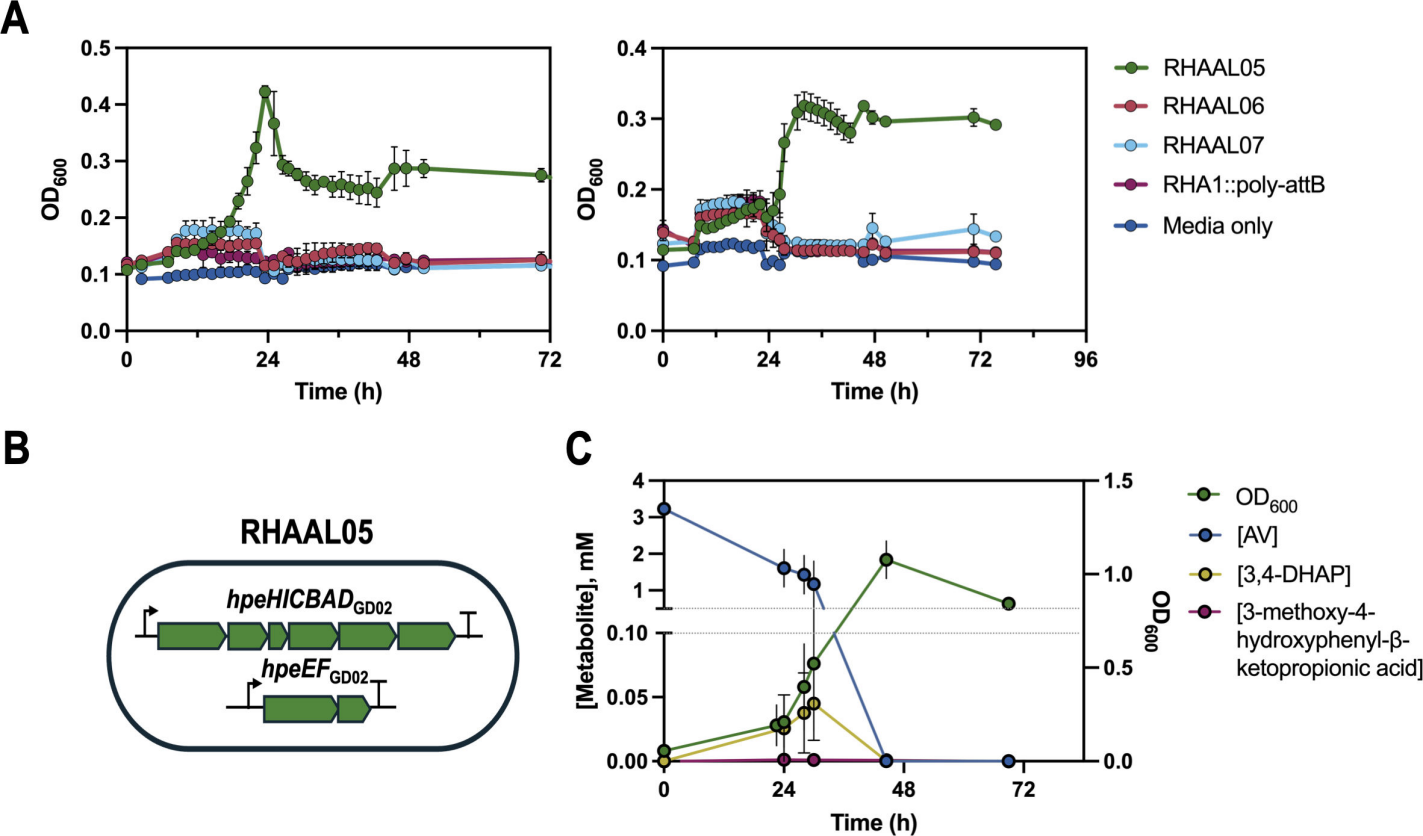
Growth of HPE pathway strains on AV and HAP. (**A**) RHA1::poly-*attB* and the indicated engineered strains were grown in 48-well plates in M9 supplemented with 2 mM HAP (left) or AV (right) at 30°C. (**B**) Scheme of RHAAL05 harboring a complete HPE pathway. (**C**) Growth and metabolites of strain RHAAL05 on AV. The strain was cultivated in 125 mL flasks in M9 supplemented with 3 mM AV at 30°C. Metabolites in the culture supernatants were measured by HPLC. For panels A and C, experiments were conducted in triplicate, and error bars show standard deviation.

The Hpe pathway converts AV to vanillate and HAP to 4-hydroxybenzoate, which are growth substrates of RHA1. In contrast, AS is converted to syringate, on which RHA1 does not grow. We therefore hypothesized that the expression of the eight *hpe* genes in RHA1 would enable growth on AV and HAP. Using SAGE, we first integrated *hpeEF*, encoding the last two enzymes of the Hpe pathway of GD02, into RHA1’s genome under control of the P_T1_ promoter. This strain was subsequently transformed with pRIME plasmids encoding the upstream portions (first six genes) of the operons of GD02, SYK-6, or *A. macra*, generating strains RHAAL05, RHAAL06, and RHAAL07, respectively (Fig. 4A). Of these strains, only RHAAL05 grew on 3 mM AV and HAP (Fig. 4; Fig. S10). HPLC analysis of the supernatants of RHAAL05 cultures during growth on AV demonstrated complete substrate depletion within 48 h. The concentration of 3,4-DHAP in the culture medium reached a maximum of 67 µM, representing about 2% of the initial concentration of AV (Fig. 4C). Trace amounts of 3-methoxy-4HPβKP were also detected.

To evaluate expression strategies, we also generated strain RHALR01, which expresses the entire *hpeHICBADEF* operon from GD02 as a single transcriptional unit under the control of P_T1_ in pRIME. Strains RHALR01 and RHAAL05 grew at similar rates on benzoate, HAP, and AV (Fig. S11A). Consistent with these observations, RHALR01 accumulated similar levels of pathway intermediates as RHAAL05 during growth on AV (Fig. S11B). These data indicate that expressing the full operon in a single transcriptional unit provides no apparent advantage under the conditions tested.

**Fig 5:**
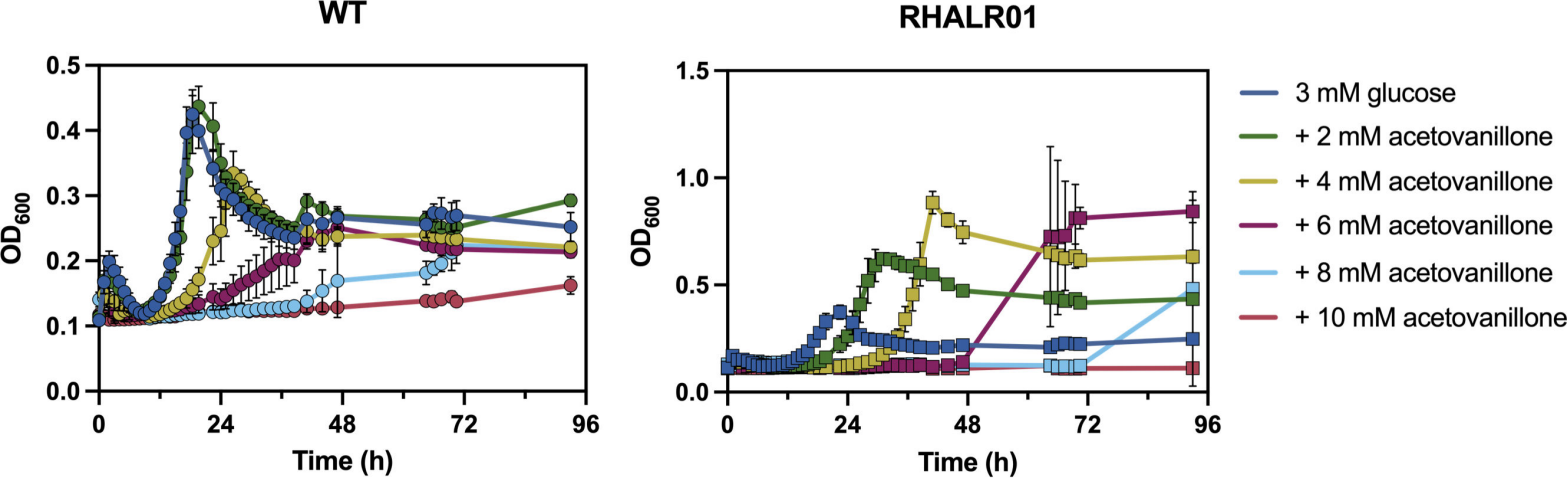
Tolerance of RHA1 WT and RHALR01 to AV. Growth of WT and RHALR01 on glucose with increasing concentrations of acetovanillone. RHA1 WT and RHALR01 were grown in 3 mM glucose M9 containing the indicated concentration of AV in 48-well plates at 30°C. Experiments were performed in triplicate. Error bars show standard deviations. Growth rates are reported in Table S4.

**Fig 6:**
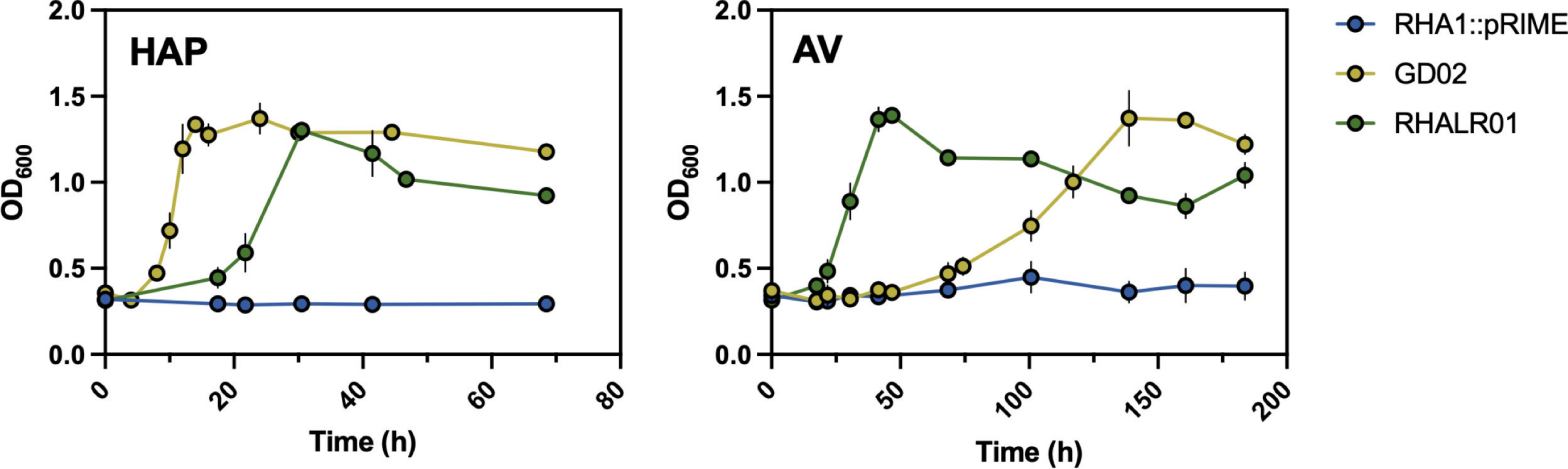
Growth of GD02 and RHALR01 on AV and HAP. Strains were grown in M9 supplemented with 3 mM HAP (left) or AV (right) in 125 mL flasks at 30°C. Experiments were performed in triplicate. Error bars show standard deviations. Growth rates of RHALR01 were 0.084 (± 0.01) and 0.053 (± 0.003) h^−1^ on HAP and AV, respectively. Growth rates of GD02 were 0.23 (± 0.02) and 0.015 (± 0.0002) h^−1^ on HAP and AV, respectively.

To further characterize RHALR01, we compared its tolerance to AV with that of WT RHA1. The two strains were grown on 3 mM glucose and different concentrations of AV. At 2 mM, AV did not significantly affect the growth of WT RHA1 on glucose. Above 2 mM, AV increased lag times and decreased growth rates in a concentration-dependent manner, with no significant growth observed in the presence of 10 mM AV after 4 days (Fig. 5; Table S4). Interestingly, RHALR01 did not tolerate higher concentrations of AV than WT, but grew at faster rates and to higher density on 3 mM glucose with 4 mM AV, consistent with its ability to catabolize AV. Interestingly, WT grew faster than RHALR01 on glucose alone, suggesting that the constitutive expression of the *hpe* operon imposes a metabolic burden (Fig. 5; Table S4). Finally, in comparing the growth rates of RHALR01 and GD02, both strains grew faster on HAP than on AV. However, GD02 grew faster than RHALR01 on HAP, while RHALR01 grew faster than GD02 on AV (Fig. 6). To determine whether this difference could be attributed to the strains’ respective abilities to catabolize vanillate, we compared their growth on this substrate. Interestingly, GD02 grew on vanillate with a significantly shorter lag than RHA1 (Fig. S12).

## DISCUSSION

HPEs, including AV and HAP, are major aromatic constituents of several lignin streams, including those generated via oxidative catalytic fractionation and Kraft pulping (8–13). This study describes a cometabolic pathway in RHA1 that is induced by and partially transforms HPEs, but does not support growth. Targeted gene deletion established that the HPEs are cometabolized by the pathway that catabolize alkylated guaiacols and phenols. Specifically, AgcAB catalyzes the *O*-demethylation of AV, while AphAB catalyzes the hydroxylation of HAP. Moreover, AphC cleaves the resulting catechol, 3,4-DHAP. The activities of AgcAB and AphC were validated by enzymatic studies, expanding the known substrate range of these enzymes. Both the *O*-demethylation and *meta*-cleavage activities were induced by AV. Finally, integration of the *hpe* genes of GD02 into RHA1 conferred growth on AV and HAP with no major metabolic bottleneck. Homologous pathways from SYK-6 and *A. macra* sp. NBRC-14102 also enabled RHA1 to transform these compounds, but at rates insufficient to support growth.

The relatively high apparent specificity of AgcA for AV (*k*_cat_/*K*_M_ = 7 ± 3 mM^−1^s^−1^) and the high degree of coupling of this reaction (~90%) are consistent with the enzyme’s high specificity for 4-substituted guaiacols, such as 4-PG (*k*_cat_/*K*_M_ = 97 ± 12 mM^−1^s^−1^). This preference for 4-substituted substrates distinguishes AgcA from two other CYP255A subfamily members, GcoA and SyoA, whose preferred substrates are guaiacol and syringol, respectively (38, 39). Consistent with these substrate specificities, GcoA has low activity on vanillin, while SyoA does not bind AS and has low affinity for syringaldehyde (39, 40). In agreement with our *in vivo* data, AgcA has very low specific activity for AS (36). Structural studies of AgcA_EP4_, which shares 58% amino acid sequence identity with AgcA_RHA1_, have revealed that the 4-alkyl moiety is accommodated by a hydrophobic cavity lined by Ala293 and Leu78 (36). In GcoA and SyoA, Ala293 corresponds to Thr296 and Ser300, respectively, residues with larger side chains that fill the cavity. Moreover, the catalytic versatility of P450s and their amenability to protein engineering (41) suggests that this previously unreported *O*-demethylation of AV could readily be enhanced.

The relatively high specific activity of AphC for 3,4-DHAP is similarly consistent with this extradiol dioxygenase’s preference for 4-substituted catechols (30). Like AgcA, AphC has a hydrophobic cavity that can accommodate a range of 4-substituents, with the specific activity of AphC for 4-methyl and 4-phenyl catechols only differing by a factor of two (30). Interestingly, the enzyme’s specific activity for 4-chlorocatechol is 8% that of 4-methylcatechol, which is remarkably similar to that for 3,4-DHAP. This may reflect the electron-withdrawing effects of these substituents on the catechol.

The degradation of AV and HAP by RHA1 represents a novel example of cometabolism, wherein the fortuitous activities of AgcA, AphA, and AphC result in the partial degradation of the HPEs. Although AV induced both *O*-demethylation and *meta*-cleavage activities, the accumulation of a bright yellow color in the supernatants of RHA1 cultures incubated with AV suggests that the 3,4-DHAP *meta*-cleavage product is not further metabolized. Indeed, no metabolites predicted to be produced by downstream Aph enzymes were detected. This indicates that AphD, the *meta*-cleavage product dehydrogenase (31), either does not significantly transform the 3,4-DHAP *meta*-cleavage product or that *aphD* is not significantly expressed under these conditions. In EP4, *agcAB*, *aphC*, and *aphHID* occur in three putative operons, as inferred by transcriptomic data during growth on 4-propylguaiacol (32). In both EP4 and RHA1, the *aph*/*agc* gene cluster harbors two putative AraC-type transcriptional regulators, RS18810 (annotated as AphR) and RS18790. These share 99.4% and 47.8% amino acid sequence identity, respectively, with NphR, a transcriptional activator involved in the catabolism of 4-nitrophenol (42). The structural similarity between AV and alkylguaiacols suggests that the former, or its metabolic derivatives, may be able to interact with RS18810 and/or RS18790. Finally, the biocatalytic potential of these cometabolic activities is highlighted by the pharmacological properties of 3,4-DHAP. The latter is produced by certain plants and possesses anti-inflammatory, antimelanogenic, antioxidative, and cardioprotective properties (43–45). Indeed, a microbial cell factory based on *E. coli* has been engineered to produce 3,4-DHAP from 1-(4-hydroxyphenol)-ethanol (46).

Comparison of the efficacy of three homologous Hpe pathways in RHA1 provides important insights into aspects of pathway function in this host as well as a basis for optimizing biocatalyst design. The significantly higher functionality of the GD02 pathway, as determined by production of the HPβKPs, is consistent with the strain of origin and host belonging to the same genus. Considering that the expression of the three sets of genes was driven by the same promoter, this suggests that translation signals and/or post-translational factors contribute to the observed activities. Interestingly, the substrate preference of the GD02 pathway in RHA1 mirrors the substrate specificity of HpeHI, the kinase that initiates HPE catabolism. More particularly, the substrate specificities (*k*_cat_/*K*_M_) of HpeHI for AV and HAP are quite similar, and at least 22× higher than for AS (18). By comparison, the GD02 pathway transformed AV and HAP to their corresponding HPβKPs at similar rates and over 1,000× faster than AS (Table 4). Despite these similarities between *in vitro* and *in vivo* specificities, both GD02 and RHA1 harboring the GD02 pathway grew significantly faster on HAP than on AV, suggesting that other factors contribute to growth on these substrates. In contrast, the substrate preference of the SYK-6 pathway in RHA1 reflects neither the substrate specificity of the initial kinase, AcvAB, nor the growth rates of SYK-6 on the different HPEs. More particularly, AcvAB had the highest specific activity for HAP, with those of AV and AS being 10 and 17%, respectively, of this value. More importantly, SYK-6 grows faster on AS than AV (15). In contrast, our experiments indicate that in RHA1, the pathway has similar activity for AV and HAP, and that this is ~6-fold higher than for AS. Importantly, differences in substrate preference may be impacted by factors other than the kinase’s specificity such as the gene expression, protein production levels, and the cellular environment. For example, the accumulation of phosphorylated HPEs in RHAAL03 (Fig. S9) suggests that the phosphophenylethanone carboxylase of SYK-6 may not be efficiently produced in RHA1.

The Hpe pathway of GD02 was the only one of the three that enabled the growth of RHA1 on AV and HAP, consistent with the significantly higher activity of the GD02 pathway in resting cell assays. Although *O*-demethylation is often rate-limiting in biocatalytic processes (47, 48), vanillate did not accumulate during the growth of RHALR01 on AV. The stoichiometric generation of formaldehyde in the *O*-demethylation of vanillate may contribute to the slower growth on AV compared to HAP. In this respect, genes encoding a putative mycothiol-dependent formaldehyde detoxification pathway were upregulated in GD02 during growth on AV (16). Interestingly, RHALR01 grew faster on AV than GD02, in contrast to GD02 growing faster than RHALR01 on HAP and vanillate. GD02 carries two copies of the *vanAB* genes, which may increase the rate of vanillate catabolism (16). The faster growth of RHALR01 on AV may reflect differences in *hpe* pathway expression levels in this strain versus GD02. In GD02, the *hpe* genes were upregulated to lower levels during growth on AV compared to HAP, as well as to lower levels than the *vanAB* genes (16). Other factors that may contribute to the differences in growth phenotypes include variations in rates of substrate diffusion across the membrane and in tolerance levels. Nevertheless, expression of the *hpe* genes did not enhance RHA1’s tolerance to AV, instead increasing lag times and decreasing growth rates in the absence of AV. The Hpe pathway requires the synthesis of eight proteins and utilizes three molar equivalents of ATP per aromatic, which could represent a metabolic burden on the cell. Strategies such as adaptive laboratory evolution (ALE) could help reduce lag times and improve strain performance (49).

The transformation of AV and HAP by AgcAB and AphAB raises questions about this pathway’s compatibility with Hpe pathways. Genomic analysis indicates that none of GD02, SYK-6, and *A. macra* have homologs of *agcA* or *aphA*. However, *Rhodococcus* sp. JVH1 (50) harbors both a complete, syntenous *hpe* operon as well as an *aph*/*agc* cluster. JVH1 is highly similar to RHA1 (ANI 98.6%). However, it is unknown whether JVH1 grows on HPEs or 4-alkylated phenols and guaiacols. Catabolic segregation may be enabled through tighter regulatory mechanisms. In RHA1, the physiological significance of the induction of the *aph* and *agc* genes by AV and HAP is unclear. Importantly, it is possible that the naturally occurring concentration of these HPEs is too low to induce the expression of the *aph* and *agc* genes in these strains.

During the growth of RHALR01 on AV, about 2% of the initial carbon was shunted to AgcA. The ability of HpeHI to outcompete AgcAB could be due to differences in enzyme production levels and/or their specificities for AV. From a metabolic engineering perspective, deleting *agcA* could increase product and biomass yields. More broadly, the characterization of the native and engineered catabolism of HPEs in RHA1 described here broadens the range of commodity chemicals that could be accessed by microbial cell factories and facilitates the latter’s development to valorize these substrates.

## MATERIALS AND METHODS

### Chemicals and reagents

Reagents were obtained from Sigma-Aldrich and Fisher Scientific and were of analytical grade unless otherwise noted. Enzymes for DNA amplification and manipulation were purchased from New England Biolabs. Oligonucleotides were synthesized by Integrated DNA Technologies.

### DNA manipulations and genomic insertions

Plasmids and oligonucleotides used in this study are listed in Tables S1 and S2, respectively. Operons were amplified from genomic DNA isolated from *R. rhodochrous* GD02 (NZ_CP083974.1), *S. lignovorans* SYK-6 (NC_015976.1), or *A. macra* NBRC 14102 (NZ_BCQT01000001.1) using Q5 DNA polymerase. The GD02 and *A. macra* operons were amplified as a single PCR product, while the SYK-6 operon was amplified as two products (*acvAB* and *acvCDEF*) using primers listed in Table S2. Plasmids were assembled using NEBuilder HiFi DNA Assembly Master Mix according to the manufacturer’s recommendations (51) and propagated in *E. coli* DH5α pir. Electrocompetent cells of *E. coli* and RHA1 were prepared by washing freshly grown cells thrice in ice-cold water and once in 10% glycerol before flash-freezing in liquid nitrogen. Cells were transformed using a Bio-Rad MicroPulser at 2.0 kV for 5 ms. Plasmid sequences were validated by sequencing (Plasmidsaurus). For RHA1 genomic integrations using pRIME, strains were electroporated with the corresponding plasmid and rescued on LB with 30 μg/mL apramycin, and transformants were verified by colony PCR. pAL01 integration using SAGE was performed as described previously (27). Briefly, genes were cloned into the pJE1828 cargo vector using primers listed in Table S2, and the resulting plasmid was co-electroporated with its corresponding integrase-expressing plasmid, pGW31, into RHA1 poly-*attB*. Competent cells of the resulting sucrose-sensitive, kanamycin-resistant clones were subsequently transformed with pJE1817 to excise the cargo plasmid backbone. Backbone excision and site-specific insertion in sucrose-resistant, kanamycin-sensitive clones were confirmed by colony PCR.

### Growth of bacterial strains

*Rhodococcus* strains used in this study are listed in Table 5. For growth on minimal media, RHA1 and GD02 were cultivated in M9 medium supplemented with Goodies (52) at 30°C with shaking at 200 rpm. Aromatic substrates were dissolved in dimethyl sulfoxide (DMSO) and added to the growth medium. Seed cultures were grown in LB supplemented with appropriate antibiotics: 50 μg/mL apramycin sulfate for RHA1-carrying pRIME plasmids and 100 μg/mL ampicillin for *E. coli*-carrying pRIME plasmids. Cultures were grown in 96-well plates containing 150 μL of media or in 125-mL flasks containing 25 mL of media. OD_600_ was measured in a Tecan Spark 20 M (for Fig. 4B and 5) or VersaMax (for Fig. 6) microplate reader using a 96-well plate in a Biowave II spectrophotometer using cuvettes (for Fig. 4C).

**TABLE 5:** Strains used in this study

Strain	Description	Source
*E. coli* DH5α PIR	DNA propagation	(53)
*E. coli* BL21	Protein production	Invitrogen
*E. coli* S17-1	Used to conjugate pk18 plasmids with RHA1	(54)
WT RHA1	*Rhodococcus aromaticivorans* RHA1	(55)
WT GD02	*Rhodococcus rhodochrous* GD02	(8)
RHA1::poly*-attB*	RHA1 with 10x poly-*attB* site integrated at *RS20555*	(27)
RHA1 *agcA*	RHA1 *agcA* knockout	(32)
RHA1 *pobA*	RHA1 *pobA* knockout	(34)
RHA1 *vanA*	RHA1 *vanA* knockout	(33)
RHA1 *aphA*	RHA1 *aphA* knockout	(31)
RHA1::pRIME	RHA1 with empty pRIME vector	This study
RHAAL01	RHA1 poly-*attB*::*hpeEF*_GD02_	This study
RHAAL02	RHA1 poly-*attB*::pRIME-*hpeHICBAD*_GD02_	This study
RHAAL03	RHA1 poly-*attB*::pRIME-*acvABCDEF*_SYK-6_	This study
RHAAL04	RHA1 poly-*attB*::pRIME-*hpeHICBAD*_A.macra_	This study
RHAAL05	RHA1 poly-*attB*::*hpeEF*_GD02_::pRIME-*hpeHICBAD*_GD02_	This study
RHAAL06	RHA1 poly-*attB*::*hpeEF*_GD02_::pRIME-*acvABCDEF_SYK-6_*	This study
RHAAL07	RHA1 poly-*attB*::*hpeEF*_GD02_::pRIME- *hpeHICBAD*_A.macra_	This study
RHAAL08	RHA1 poly-*attB*::*hpeEF*_GD02_::pRIME	This study
RHALR01	RHA1::pRIME-*hpeHICBADEF*_GD02_	This study

### Protein production and purification

AgcA_RHA1_ and AgcB_EP4_ were produced heterologously as N-terminal polyHis-tagged proteins and purified using immobilized metal affinity chromatography as previously described (32), with slight modifications (36). AphC was produced and purified as described in reference 30.

### Measurements of *O*-demethylation activity

Specific activity and apparent steady-state kinetics were determined as described previously (35) with minor modifications. Briefly, for specific activity, 1 µM AgcAB, 100 µM substrate, and 1,000 U/mL catalase were combined in 10 mM MOPS, pH 7.2 (ionic strength [*I*] = 25 mM) in 200 µL cuvettes. The buffer was prepared by titrating 5 mM NaOH with MOPS to a pH of 7.2 (~10 mM MOPS, *I* = 5 mM), then adding NaCl to 20 mM. NADH was added to 200 µM to initiate the reaction, then its depletion was monitored by absorbance at 340 nm, ε = 6.22 mM^−1^ cm^−1^ (Cary 60). After ~3 min, the reaction was quenched by the addition of 10% acetic acid, and the remaining concentration of the aromatic was determined by HPLC separation, UV detection, and interpolation of a standard curve of authentic standards (0–200 µM). Apparent steady-state kinetic parameters of AgcAB were determined by the same assay except a range of substrate concentrations (2.5–250 µM) was used, and AgcB was added to initiate the reaction. Reaction rate was determined by depletion of NADH, corrected for baseline (1 µM AgcB oxidation of NADH) and coupling (average coupling for 100 µM calculated in the above assay). Apparent steady-state kinetic parameters were calculated by fitting Michaelis–Menten equations to the initial velocity of reactions at various concentrations of aromatic substrate (Graphpad PRISM, version 10.0). As previously described, AgcB from *R. rhodochrous* EP4 (63% amino acid sequence identity to AgcB_RHA1_) was used as the reductase in these assays as it transfers electrons efficiently to AgcA_RHA1_ and is more soluble than AgcB_RHA1_ (32).

For measuring *O*-demethylation activity in AV or EG-induced cells, Δ*aphC* or Δ*agcA* cells were grown in 60 mL LB to mid-log (OD_600_ 0.2–0.6), washed twice in M9, and suspended in 1 mL M9 in a 24-well plate to an OD of 1.0. Cultures were incubated for 2.5 h with 1 mM AV, EG, or without substrate to allow for gene induction before being centrifuged, washed, and suspended in fresh media with 1 mM AV. Supernatants were sampled after 0, 30, and 60 min of incubation at 30°C with shaking. Samples were acidified by adding 10% glacial acetic acid, centrifuged, and stored at −20°C until analysis by HPLC. For data normalization, culture pellets were lysed by bead-beating, lysates were spun down to remove debris, and total protein was measured using a Thermo Scientific Pierce BCA Protein Assay Kit according to the manufacturer’s instructions.

### Measurements of *meta*-cleavage activity

The specific activity of AphC was measured by monitoring oxygen consumption using a Clark-type polarographic O_2_ electrode OXYG1 (Hansatech) as described in reference 30. Assays were performed using air-saturated 20 mM HEPES (36) (*I* = 0.1 M, pH 8.0) at 25°C, and reactions contained 1.4 μM (for 3,4-DHAP) or 0.46 μM (for 4-methylcatechol) AphC and 200 μM catechol. Dioxygenase induction was determined by spectrophotometric measurements of *meta*-cleavage activity in lysates. RHA1 was grown in 0.1% glucose to mid-exponential, and pellets were collected by centrifugation and washed in M9. Cells were suspended in M9, and AV, EG, or succinate was added to 1 mM. Cultures were incubated a further 7 h at 30°C with shaking. Pellets were later thawed, lysed by bead-beating, and clarified by centrifugation. *meta*-Cleavage-specific activity in lysates was measured as described in reference 30, and data were normalized to total protein measured as described above.

### Resting cell assays

Strains were grown in LB to early-mid log (OD_600_ 0.2-1). Cells were harvested, washed in M9 with 0.1% glucose, and suspended in the same medium to the desired OD_600_. For comparison of the engineered HPE-degrading strains, cells from 100 mL initial LB cultures were suspended in 1 mL M9 and added to 24-well plates. Assays were initiated by the addition of substrate to 1 mM. For screening RHA1 mutants for AV and HAP transformation, strains were grown in 150 mL LB, and cells were suspended in 5 mL cultures in flasks. Assays were initiated by the addition of substrate to 1 mM. To assess the depletion of 4-methylcatechol by RHA1 under different induction conditions, cells from 100 mL LB were suspended in 2 mL M9 in culture tubes with 1 mM AV or 4-propylphenol or 2 mM or succinate. Cells were incubated for 3 h, harvested, washed, and suspended in fresh medium with 0.25 mM 4-methylcatechol. For all resting cell assays, flasks, tubes, or plates were incubated at 30°C with shaking, and aliquots were sampled at regular timepoints, spun down, and stored at −20°C until analysis. Calculated rates were normalized to total protein concentration in the initial suspensions, measured as described above. Assays were conducted in triplicate.

### HPLC analysis

For detection and quantification of aromatic compounds, culture supernatants were centrifuged at 16,000 × *g* for 10 min and run over a Luna 5 µm C18 100 Å 150 × 3 mm column (Phenomenex) by a Waters 2695 separation module. The flow rate was 0.7 mL min^−1^, and the samples were eluted with a linear gradient from 0.1% acetic acid in water with 1% methanol to 100% methanol. Compounds were detected by a Waters 2669 photodiode array detector at a wavelength of 280 or 260 nm. Concentrations of 3,4-DHAP, AV, HAP, and AS were determined by interpolating a standard curve of authentic standards. For quantification of β-ketopropionate products, concentrated cells of RHAAL02 were incubated with 3 mM HPE until complete turnover was achieved, as verified by HPLC. The product was then isolated by semi-preparative HPLC and used to prepare serial dilutions. The samples were incubated at room temperature overnight to allow for spontaneous decarboxylation of the product to the HPE. Decarboxylated products were verified by HPLC to ensure that all the carboxylated substrates were depleted, and that only the HPE was produced. Standard curves for the β-ketopropionate products were thus generated and utilized to quantify compounds in resting cell assays.

### LC-MS analysis

For detection of pathway metabolites, culture supernatants were centrifuged at 16,000 × *g* for 10 min and analyzed by LC-MS using an Agilent 1290 Infinity II UHPLC in line with an Agilent 6546 Q-TOF equipped with a dual AJS ESI source operating in positive and negative ionization modes. A sample (2 µL) was injected onto a Zorbax Eclipse Plus C18 Rapid Resolution HD, 2.1 × 50 mm 1.8 µm column and run on a 20 min linear gradient from 5% to 100% solvent B at 0.45 mL min^−1^. Solvent A was 0.1% formic acid in water, and solvent B was 0.1% formic acid in methanol. MS parameters were as follows: capillary voltage, 3,500 V; nozzle voltage, 500 V; drying gas temp, 300°C; drying gas flow rate, 10 L min^−1^; sheath gas temperature, 350°C; sheath gas flow rate, 12 L min^−1^; nebulizer pressure, 45 psi; and fragmentor voltage, 100 V. The MS parameters used for negative mode were the same as positive with the following differences: capillary voltage, 4,000 V; nozzle voltage, 2,000 V. MS/MS was collected on selected ions with 10, 20, and 40 V collision energies. Data were collected and analyzed using MassHunter Workstation version 10.

## Data Availability

Additional data can be found in the supplemental material.

## SUPPLEMENTAL MATERIAL

Supplemental material aem.02522-25-s0001.docx Tables S1 to S4; Fig. S1 to S12.

ASM does not own the copyrights to Supplemental Material that may be linked to, or accessed through, an article. The authors have granted ASM a non-exclusive, world-wide license to publish the Supplemental Material files. Please contact the corresponding author directly for reuse.
