# Supplementary material for: Characterization of acetovanillone degradation in wild-type and engineered Rhodococcus aromaticivorans RHA1
Source: Appl Environ Microbiol. 2026 Mar 20;92(4):e02522-25. doi: 10.1128/aem.02522-25 (PMC13101465; doi:10.1128/aem.02522-25)
Supplement: Supplemental material — Tables S1 to S4; Fig. S1 to S12. [file aem.02522-25-s0001.docx]

**Table S1. Plasmids used in this study**

| PLASMID | Description^a^ | Source |
| --- | --- | --- |
| pRIME | $\varphi$C31, R6K, *bla, aac(3)IV* | (1) |
| pAL02 | pRIME- P _T1_-*hpeHICBAD*_GD02_ | This study |
| pAL03 | pRIME- P_T1_-*acvABCDEF*_SYK-6_ | This study |
| pAL04 | pRIME- P_T1_- *hpeHICBAD_Amacra_* | This study |
| pLR01 | pRIME- P_T1_-*hpeHICBADEF*_GD02_ | This study |
| pJE1828 | *nptII*, *sacB*, BxbI *attP* | (2) |
| pAL01 | pJE1828-P_T1_-*hpeEF*_GD02_ | This study |
| pGW31 | BxbI, *aac(3)-IV* | (2) |
| pJE1817 | $\varphi$C31*, aac(3)-IV* | (2) |
| pET28a-AgcA | 10× His, TEV, *agcA*_RHA1_ | This study |
| pET28a-AgcB_EP4_ | 10× His, TEV, *agcB*_EP4_ | (3) |
| pET28a-AphC | 10× His, TEV, *aphC*_RHA1_ | (4) |

^a^Genes encode resistance against: *bla*, ampicillin; *nptII*, kanamycin; *aac(3)-IV*, apramycin.

**Table S2. Oligonucleotides used in this study**

| Oligo | Description | Sequence (5’-3’) |
| --- | --- | --- |
| oAL001 | Forward Gibson primer for constructing pAL02 | tcaggctgcgcaactgttgggaagggcgataccgctctggtcagcgac |
| oAL002 | Reverse Gibson primer for constructing pAL02 | tggcgtaatagcgaagaggcccgcaccgatactagagtcccgctgagg |
| oAL003 | Forward Gibson primer for constructing pAL03 (*acvAB*) and for screening insertion or assembly of pAL03 | ctttaagaaggagatatacatatgagcgaaccgaccaaggg |
| oAL004 | Reverse Gibson primer for constructing pAL03 (*acvAB*) and for screening insertion or assembly of pAL03 | gtgaggctcatcttatctgcgtcccccgaaaatc |
| oAL005 | Forward Gibson primer for constructing pAL03 (*acvCDEF*) | acgcagataa*g*atgagcctcacggcaaag |
| oAL006 | Reverse Gibson primer for constructing pAL03 (*acvCDEF*) | cacgggtgccggtgggtcgactagttcagcccagcaggccgag |
| oAL007 | Forward Gibson primer for constructing pAL04 | tgtttaactttaagaaggagatatacatatgaccaccgcagacggcgag |
| oAL008 | Reverse Gibson primer for constructing pAL04 | ggctcacgggtgccggtgggtcgactagtgtcctctgcacctactg |
| oLR001 | Forward Gibson primer for constructing pLR01 (*hpeHI*) | ttgtttaactttaagaaggagatatacatatgacgatcaccgagagc |
| oLR002 | Reverse Gibson primer for constructing pLR01 (*hpeHI*) | gtcggtgctcatcgggtctgtcccttcgctgc |
| oLR003 | Forward Gibson primer for constructing pLR01 (*hpeCBA*) | cgaagggacagacccgatgagcaccgacaccac |
| oLR004 | Reverse Gibson primer for constructing pLR01 (*hpeCBA*) | gagttcgtcgcggttcactgggttcctccggtg |
| oLR005 | Forward Gibson primer for constructing pLR01 (*hpeDEF*) | gaggaacccagtgaaccgcgacgaactccttac |
| oLR006 | Reverse Gibson primer for constructing pLR01 (*hpeDEF*) | gaggggctcacgggtgccggtgggtcgactagttagtgtttgatgccgagaac |
| oAL009 | Forward Gibson primer for constructing pAL01 from pRIME-P_T1_-*hpeEF*_GD02_ | tttggtccggatccgatatcgccaagcttgggctgcag |
| oAL010 | Reverse Gibson primer for constructing pAL01 from pRIME-P_T1_-*hpeEF*_GD02_ | tgttcgtcctcgagtctagaactagagtcccgctgagg |
| oAL011 | Forward screening primer for verifying insertion or assembly of pAL02 and pLR01 | tttaactttaagaaggagatatacatatgacgatcaccgagagcaag |
| oAL012 | Reverse screening primer for verifying insertion or assembly of pAL02 and pLR01 | gtgtcggtgctcatcgggtctgtcccttc |
| oAL013 | Forward screening primer for verifying insertion or assembly of pAL04 | tcgctactggacgaagagga |
| oAL014 | Reverse screening primer for verifying insertion or assembly of pAL04 | tgtaggttcccacgtgttcg |
| oAL015 | Forward screening primer for verifying insertion of pAL01 (flanking BxbI *attB* site) | tgatcgaattctttcatttaagaccct |
| oAL016 | Reverse screening primer for verifying insertion of pAL01 (flanking BxbI *attB* site) | ggcagaattttgggagtggcat |

**Table S3. Specific activity of AgcA^a^.**

| **Substrate** | **Substrate depletion** | **NADH depletion** | **Coupling** |
| --- | --- | --- | --- |
|  | min^-1^ | min^-1^ | % |
| 4-EG | 40 (3) | 38 (2) | 110 (7) |
| AV | 4.5 (0.5) | 5 (0.4) | 90 (10) |

^a^Values were measured using 100 µM aromatic substrate, 175 µM NADH, 1 µM AgcAB, 100 U/mL catalase in 10 mM MOPS, pH 7.2, *I* = 25 mM, 25 °C. Aromatic substrate depletion was measured by HPLC and NADH depletion by spectrophotometry.

**Table S4. Growth rates of RHA1 WT and RHALR01 on AV with glucose.**

|  | **Growth rate^a^** | |
| --- | --- | --- |
| **[AV]** | **RHA1 WT** | **RHALR01** |
| mM | h^-1^ | h^-1^ |
| 0 | 0.19 (0.02) | 0.13 (0.01) |
| 2 | 0.17 (0.02) | 0.15 (0.01) |
| 4 | 0.08 (0.009) | 0.17 (0.01) |

^a^Strains were grown in 3 mM glucose M9 supplemented with the indicated concentration of AV in 48 well plates at 30 °C. Growth rates were calculated using at least three consecutive points. Values represent average of triplicates with standard deviation in parentheses.

**Figure S1**. Identification of 3,4-DHAP as the transformation product of AV and HAP by RHA1. Overlayed EICs (**A**) and mass spectra (**B**) of a 3,4-DHAP standard with the product of incubations of RHA1::pRIME with AV and HAP.

**Figure S2.** Incubation of RHA1 with AS. RHA1::pRIME was incubated in 10 mL M9 containing 0.1% glucose and 1 mM AS in a 25-mL flask at 30 °C. HPLC chromatograms show culture supernatants analyzed after 0 and 4 h of incubation. No significant difference in peak areas was detected. AS was identified based on its match to an authentic standard.

**Figure S3.** Transformation of AV and HAP by select RHA1 gene deletion mutants. RHA1::pRIME and the incubated mutant strains were incubated in 10 mL M9 containing 0.1% glucose and 1 mM AV (left) or HAP (right) in 25 mL flasks at 30 °C. HPLC chromatograms show culture supernatants analyzed after 0 and 3, 4 or 5 h of incubation. The proposed transformations of AV (**1**) and HAP (**3**) to 3,4-DHAP (**2**) are shown. Construction of the mutant strains is described in the following publications: Δ*vanA* (5), Δ*agcA* (3), Δ*pobA* (6), and Δ*aphA* (7).


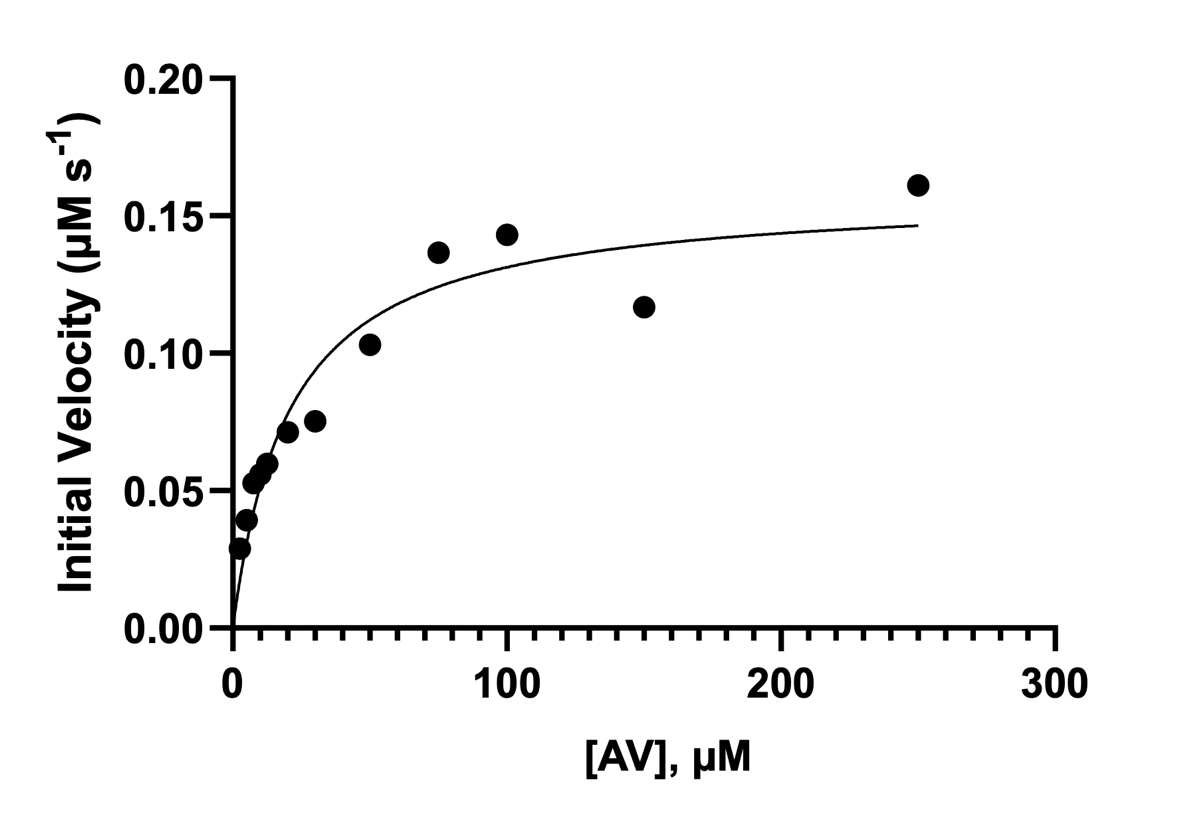


**Figure S4.** Steady-state kinetic analysis of AgcA. Rates were measured using 1 µM AgcAB and 175 µM NADH in 10 mM MOPS, pH = 7.2, *I* = 25 mM at 25 °C. NADH depletion was monitored spectrophotometrically. The curved line represents a best fit of the Michaelis-Menten equation to the data.

**Figure S5.** Modeled structure of the AgcA:AV complex. Two views of the binding pocket with the pocket surface, highlighting possible steric clashes. AV (green) is modeled in its coplanar conformation (**A**, **C**) or with a freely rotating aryl-acetyl C-C bond (**B**, **D**) overlayed onto 4-EG (magenta). Atomic distances between the AV carbonyl oxygen and the indicated atom are illustrated (dashed line). The view in panels C and D was made by rotating the view in panels A and B 90$^{\circ}$ about the *y*-axis. AgcA_RHA1_ was generated using SWISS-MODEL (8) using AgcA_EP4_ (PDB 9IA1). Heme and 4-EG are positioned as in the crystal structure of the AgcA_EP4_:4-EG complex. AV was manually overlayed onto 4-EG based on the guaiacyl core. PyMOL (9) was used for model visualization.

**Figure S6.** Accumulation of a yellow color in RHA1 cultures. Concentrated cells of RHA1 WT were incubated overnight with M9 medium containing 1 mM AV (**A**), HAP (**B**) or glucose (**C**) in 48 well plates at 30 °C.

**Figure S7**. LC-MS characterization of the *meta*-cleavage product of 3,4-DHAP. RHA1::pRIME was incubated overnight at 30 °C in M9 supplemented with 0.1% glucose and 1 mM AV. The EIC (left) corresponding to the expected *m/z* of the *meta*-cleavage product and its corresponding mass spectrum (right) are shown. The observed *m/z* value is within 5 ppm of the theoretical value of the expected compound, indicated in the spectrum.

**Figure S8.** LC-MS characterization of the major products of RHAAL02, RHAAL03 and RHAAL04 incubated with HPEs. Strains were incubated in M9 supplemented with 0.1% glucose and 1 mM HAP (left), AV (middle) or AS (right) in 24 well plates at 30 °C. A representative extracted ion chromatogram (EIC) is shown for the major products of resting cell assays (top), and the corresponding mass spectra for each strain (bottom). The observed *m/z* values are within 5 ppm of the theoretical values for the expected 4-HPβKP, indicated in the spectra.

**Figure S9**. Accumulation of phosphorylated HPEs. The indicated strains were incubated in M9 supplemented with 0.1% glucose and 1 mM HAP (left), AV (middle) or AS (right), and the supernatants were analyzed by LC-MS after 90 min (HAP and AV) or 180 min (AS). Graphs show the area under the curve for peaks with the *m/z* values of 4-phosphoacetophenone, 4-phosphoacetovanillone or 4-phosphoacetosyringone, normalized to total protein in the incubations as well as percentage of HPE substrate consumed. Metabolite identities were validated by matching *m/z* values and retention times to those of authentic standards.

**Figure S10.** Ability of engineered RHA1 strains to grow on HPEs. Strains contained *hpeEF*_GD02_ as well as: RHAAL05, *hpeHICBAD*_GD02_; RHAAL06, *acvABCDEF*_SYK-6_; RHAAL07, *hpeHICBAD_Amacra_*; or RHA1::pRIME, empty vector control. Strains were grown in M9 supplemented with 2 mM AV (left) or HAP (right) in 48 well plates at 30 °C. Colony forming units (CFUs) were counted at inoculation and after 2 days.


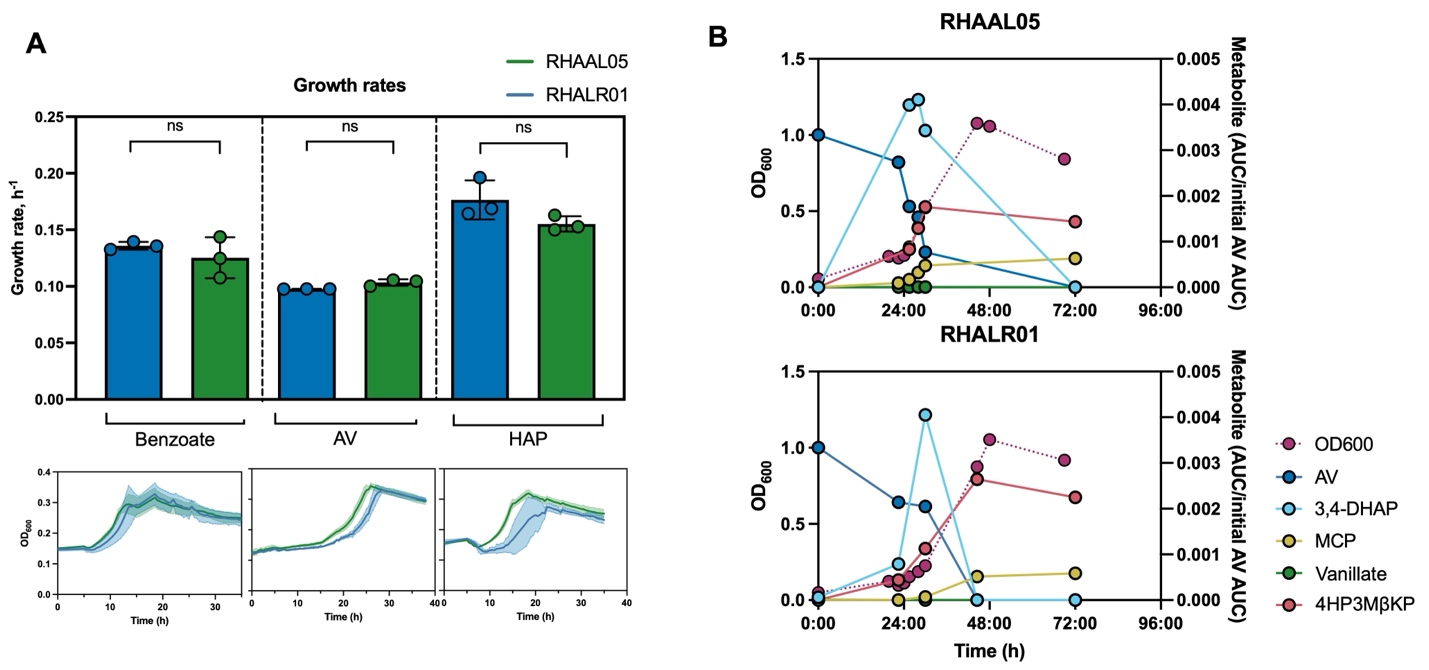


**Figure S11.** Comparison of strains RHAAL05 and RHALR01. **(A)** The growth kinetics of RHA1 expressing the *hpe* genes of GD02 as a single (RHALR01) or as two (RHAAL05) transcriptional units were compared on benzoate, AV and HAP. The strains were grown in M9 supplemented with 3 mM substrate in 48 well plates at 30 °C. **(B)** LC-MS analysis of culture supernatants of RHALR01 and RHAAL05 grown on AV. Strains were grown in 3 mM AV M9 in 125 mL flasks at 30 °C. Metabolites are represented as the area under the curve normalized to initial AV peak area. MCP, *meta*-cleavage product; 4HP3MβK, 4-hydroxyphenyl-3-methoxy-β-ketopropionate.

**
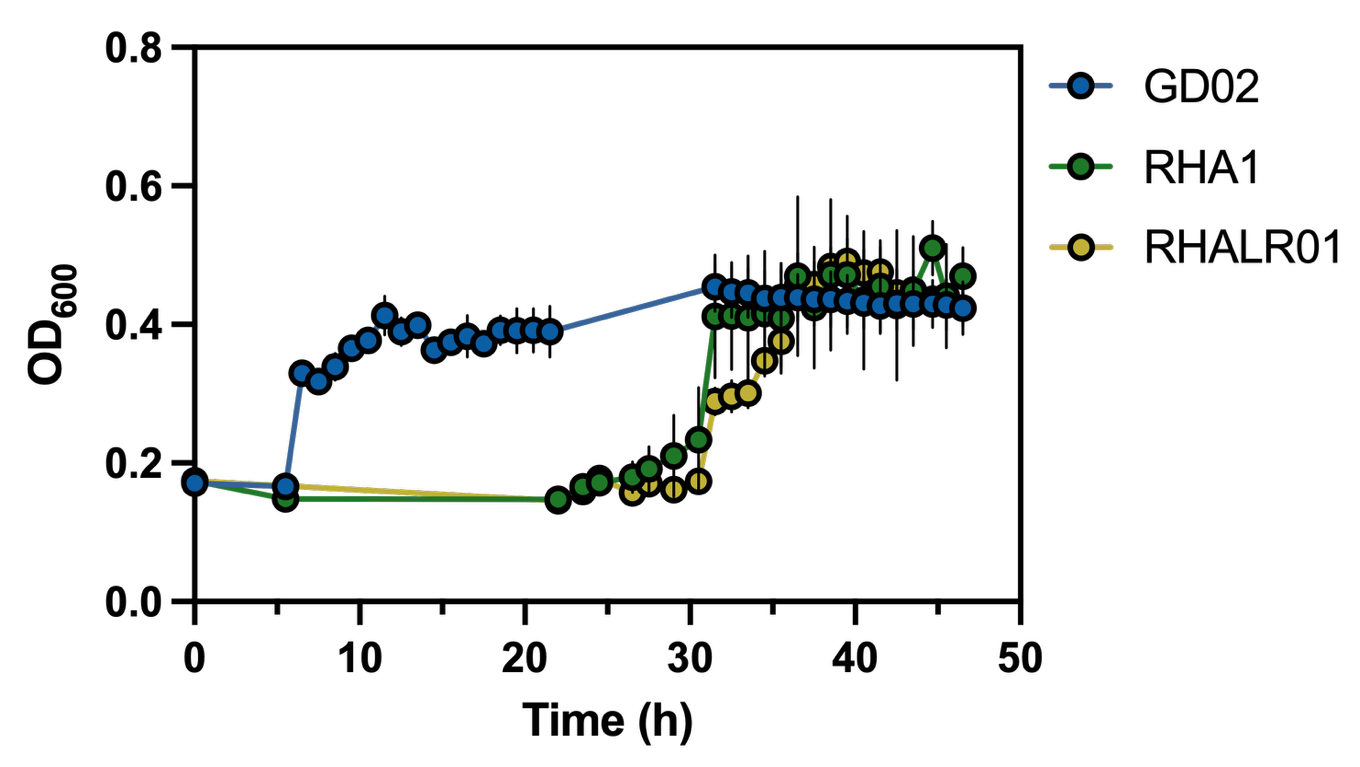
**

**Figure S12**. Growth of RHA1 and GD02 on vanillate. RHA1, RHALR01 and GD02 were grown in 3 mM vanillate M9 in 48-well plates at 30 °C. Experiments were performed in triplicate. Error bars show standard deviations.

**REFERENCES**

1. Round JW, Robeck LD, Eltis LD. 2021. An Integrative Toolbox for Synthetic Biology in *Rhodococcus*. ACS Synth Biol 10:2383-2395.

2. Elmore JR, Dexter GN, Baldino H, Huenemann JD, Francis R, Peabody GLt, Martinez-Baird J, Riley LA, Simmons T, Coleman-Derr D, Guss AM, Egbert RG. 2023. High-throughput genetic engineering of nonmodel and undomesticated bacteria via iterative site-specific genome integration. Sci Adv 9:eade1285.

3. Fetherolf MM, Levy-Booth DJ, Navas LE, Liu J, Grigg JC, Wilson A, Katahira R, Beckham GT, Mohn WW, Eltis LD. 2020. Characterization of alkylguaiacol-degrading cytochromes P450 for the biocatalytic valorization of lignin. Proc Natl Acad Sci U S A 117:25771-25778.

4. Navas LE, Zahn M, Bajwa H, Grigg JC, Wolf ME, Chan ACK, Murphy MEP, McGeehan JE, Eltis LD. 2022. Characterization of a phylogenetically distinct extradiol dioxygenase involved in the bacterial catabolism of lignin-derived aromatic compounds. J Biol Chem 298:101871.

5. Chen HP, Chow M, Liu CC, Lau A, Liu J, Eltis LD. 2012. Vanillin catabolism in *Rhodococcus* *jostii* RHA1. Appl Environ Microbiol 78:586-8.

6. Wolf ME, Lalande AT, Newman BL, Bleem AC, Palumbo CT, Beckham GT, Eltis LD. 2024. The catabolism of lignin-derived *p*-methoxylated aromatic compounds by *Rhodococcus* *jostii* RHA1. Appl Environ Microbiol 90:e0215523.

7. Levy-Booth DJ, Fetherolf MM, Stewart GR, Liu J, Eltis LD, Mohn WW. 2019. Catabolism of Alkylphenols in *Rhodococcus* via a *Meta*-Cleavage Pathway Associated With Genomic Islands. Front Microbiol 10:1862.

8. Waterhouse A, Bertoni M, Bienert S, Studer G, Tauriello G, Gumienny R, Heer FT, de Beer TAP, Rempfer C, Bordoli L, Lepore R, Schwede T. 2018. SWISS-MODEL: homology modelling of protein structures and complexes. Nucleic Acids Research 46:W296-W303.

9. Schrodinger L. 2015. The PyMOL molecular graphics system. Version 1:8.
